# Genome Sequences of Three Turkey Orthoreovirus Strains Isolated in Hungary

**DOI:** 10.1128/genomeA.01333-15

**Published:** 2015-11-19

**Authors:** Eszter Dandár, Enikő Fehér, Ádám Bálint, Péter Kisfali, Béla Melegh, Tamás Mató, Sándor Kecskeméti, Vilmos Palya, Krisztián Bányai, Szilvia L. Farkas

**Affiliations:** a United Szent István Szent László Hospital-Clinic, Budapest, Hungary; b Institute for Veterinary Medical Research, Centre of Agricultural Research, Hungarian Academy of Sciences, Budapest, Hungary; c Ceva-Phylaxia Veterinary Biologicals Co. Ltd., Budapest, Hungary; d Veterinary Diagnostic Directorate, National Food Chain Safety Office, Budapest, Hungary; e Department of Medical Genetics, Szentágothai Research Center, University of Pécs, Pécs, Hungary; f Veterinary Diagnostic Directorate, National Food Chain Safety Office, Debrecen, Hungary

## Abstract

We have investigated the genomic properties of three turkey reovirus strains—19831M09, D1246, and D1104—isolated in Hungary in 2009. Sequence identity values and phylogenetic calculations indicated genetic conservativeness among the studied Hungarian strains and a close relationship with strains isolated in the United States.

## GENOME ANNOUNCEMENT

Avian orthoreoviruses (ARVs) belong to the genus *Orthoreovirus* in the *Reoviridae* virus family*.* Their genome consists of 10 double-stranded RNA segments classified into three sizes: L (large, L1 to L3), M (medium, M1 to M3), and S (small, S1 to S4). With the exception of the S1 or S4, which encode two or three partially overlapping open reading frames, all genomic segments are monocistronic (1).

ARVs and reovirus associated diseases have been detected worldwide since the middle of the last century in commercial poultry and also in different wild-living or captive-bred bird species (2–9). Turkey reovirus (TRV) infections are usually associated with different disease manifestations including arthritis, tenosynovitis, myocarditis, and/or enteric symptoms (6, 10–13).

In this study we investigated the genomic properties of three TRV field strains isolated in Hungary in 2009. The strain 19831M was isolated from a young, 14-day-old turkey with ruffled feather, pericarditis, and enteritis, while the strains D1246 and D1104 were isolated from 35-day-old and 42-week-old healthy animals, respectively. Genome sequences of strains D1246 and 19831M09 were determined with next-generation sequencing using an Ion Torrent PGM (Life Technologies), whereas Sanger sequencing was applied in the case of strain D1104. Complete genome sequences were assembled using the CLC Genomics Workbench software (http://www.clcbio.com). Phylogenetic analysis was performed using the MEGA6 package (14).

The genomic organization of the three Hungarian TRV strains was similar and corresponded with that of the strain Reo/PA/Turkey/22342/13 isolated in the United States. The complete genome of D1104 was 23,484 bp long. Sequence termini could be determined for most of the segments except for L2 of 19831M and S3 and S4 of D1246. Each of the Hungarian TRVs encoded 12 proteins (λA, 1,293 amino acids (aa) in length; λB, 1,259 aa; λC, 1,285 aa; µA, 732 aa; µB, 676 aa; µNS, 635 aa; σA, 416 aa; σB, 367 aa; σC, 326 aa; σNS, 367 aa; p10, 99 aa; and p17, 150 aa).

The genome sequences of the three strains were most similar to each other (99.2 to 100% nucleotide and 98.2 to 100% aa identity) and were closely related to turkey strains isolated in the United States. In all gene phylogenies, the three reovirus strains were most closely related to each other and belonged to the same monophyletic group. In most gene phylogenies, the host specific evolution of the turkey, chicken, and waterfowl ARVs was evident; the only apparent exception was the gene-encoding protein µB, where chicken and turkey origin ARVs and also waterfowl origin “novel” reovirus strains clustered together, suggesting possible reassortment events in the past. In the µB gene phylogeny, the Hungarian turkey origin reovirus strains grouped together with the AVS-B (chicken) and TARV-MN4 and TERV-MN5 (turkey) strains.

As far as we know, 19831M09, D1246, and D1104 are the first characterized near-complete genomic TRV sequences from Europe. Further studies are needed to uncover the evolution and diversity of TRVs.

### Nucleotide sequence accession numbers.

The genome sequences of TRV strains 19831M09, D1246, and D1104 have been deposited in GenBank under accession numbers KR997899 to KR997908, KR997909 to KR997918, and KR997919 to KR997928, respectively.

## References

[1] Benavente J, Martínez-Costas J 2007 Avian reovirus: structure and biology. Virus Res 123:105–119. doi:10.1016/j.virusres.2006.09.005. 17018239

[2] Dandár E, Bálint Á, Kecskeméti S, Szentpáli-Gavallér K, Kisfali P, Melegh B, Farkas SL, Bányai K 2013 Detection and characterization of a divergent avian reovirus strain from a broiler chicken with central nervous system disease. Arch Virol 158:2583–2588. doi:10.1007/s00705-013-1739-y. 23771766

[3] Dandár E, Farkas SL, Marton S, Oldal M, Jakab F, Mató T, Palya V, Bányai K 2014 The complete genome sequence of a European goose reovirus strain. Arch Virol 159:2165–2169. doi:10.1007/s00705-014-2003-9. 24573219

[4] Dandár E, Huhtamo E, Farkas SL, Oldal M, Jakab F, Vapalahti O, Bányai K 2014 Complete genome analysis identifies Tvärminne avian virus as a candidate new species within the genus *Orthoreovirus*. J Gen Virol 95:898–904. doi:10.1099/vir.0.060699-0. 24421111

[5] Farkas SL, Dandár E, Marton S, Fehér E, Oldal M, Jakab F, Mató T, Palya V, Bányai K 2014 Detection of shared genes among Asian and European waterfowl reoviruses in the whole genome constellations. Infect Genet Evol 28:55–57. doi:10.1016/j.meegid.2014.08.029. 25219343

[6] Levisohn S, Gur-Lavie A, Weisman J 1980 Infectious synovitis in turkeys: isolation of tenosynovitis virus-like agent. Avian Pathol 9:1–4. doi:10.1080/03079458008418380. 18770233

[7] Olson NO 1959 Transmissible synovitis of poultry. Lab Invest 8:1384–1393. 14428822

[8] Ogasawara Y, Ueda H, Kikuchi N, Kirisawa R 2015 Isolation and genomic characterization of a novel orthoreovirus from a brown-eared bulbul (*Hypsipetes amaurotis*) in Japan. J Gen Virol 96:1777–1786. doi:10.1099/vir.0.000110. 25740958

[9] Palya V, Glávits R, Dobos-Kovács M, Ivanics É, Nagy E, Bányai K, Reuter G, Szűcs G, Dán Á, Benkő M 2003 Reovirus identified as cause of disease in young geese. Avian Pathol 32:129–138. 12745366 10.1080/030794502100007187

[10] Al Afaleq A, Jones RC 1989 Pathogenicity of three turkey and three chicken reoviruses for poults and chicks with particular reference to arthritis/tenosynovitis. Avian Pathol 18:433–440. doi:10.1080/03079458908418616. 18679874

[11] Jindal N, Mor SK, Marthaler D, Patnayak D, Ziegler AF, Goya SM, Goyal SM 2014 Molecular characterization of turkey enteric reovirus S3 gene. Avian Pathol 43:224–230. doi:10.1080/03079457.2014.904500. 24666328

[12] Mor SK, Sharafeldin TA, Abin M, Kromm M, Porter RE, Goyal SM, Patnayak DP 2013 The occurrence of enteric viruses in light turkey syndrome. Avian Pathol 42:497–501. doi:10.1080/03079457.2013.832145. 24066896

[13] Pantin-Jackwood MJ, Day JM, Jackwood MW, Spackman E 2008 Enteric viruses detected by molecular methods in commercial chicken and turkey flocks in the United States between 2005 and 2006. Avian Dis 52:235–244. doi:10.1637/8174-111507-Reg.1. 18646452

[14] Tamura K, Peterson D, Peterson N, Stecher G, Nei M, Kumar S 2011 MEGA5: molecular evolutionary genetics analysis using maximum likelihood, evolutionary distance, and maximum parsimony methods. Mol Biol Evol 28:2731–2739. doi:10.1093/molbev/msr121. 21546353 PMC3203626

